# Contribution of instant amaranth (*Amaranthus hypochondriacus* L.)‐based vegetable soup to nourishment of boarding school adolescents

**DOI:** 10.1002/fsn3.664

**Published:** 2018-06-06

**Authors:** Geoffrey Ssepuuya, James Katongole, Gaston Ampek Tumuhimbise

**Affiliations:** ^1^ Department of Food Technology and Nutrition School of Food Technology, Nutrition and Bioengineering College of Agricultural and Environmental Sciences Makerere University Kampala Uganda

**Keywords:** adolescents, amaranth, convenience foods, malnutrition, RDA

## Abstract

Uganda has one of the youngest populations in the world with about 78% of its population below 30 years of age, most of which are adolescents. The boarding school diet fed to adolescents is rich in carbohydrates and proteins but lacks adequate amounts of micronutrients to meet the adolescent RDA requirements. This study aimed at contributing to the improvement of the nutritional status of boarding school adolescents in Uganda by developing an acceptable convenient instant vegetable soup rich in protein (12.30 ± 0.25–13.26 ± 0.25%), total carotenoids (154.19 ± 12.62–292.68 ± 3.56 RAE/μg), and iron (2.33 ± 0.06–4.37 ± 0.03 mg). The three soup formulations showed desirable reconstitution and instant characteristics owing to the observed functional properties. The soup had a high dispensability (69%–71%), water absorption capacity of 1.53–1.98 g/g, bulk density of 0.79–0.80 g/ml, and swelling capacity of 5.05–5.38 g/g. The overall sensory acceptability was within a range of 6.2–6.7 and not significantly different from the control commercial soup. The soups contributed over 25% of the adolescent RDA requirements for carbohydrate, protein, dietary fiber, vitamin A, and iron but not for zinc (5.7%) and calcium (9.7%). These results indicate the potential of the soup to improve the nutritional status of adolescents. However, there is a need for additional research to increase the formulated soups’ acceptability, its contribution to zinc and calcium RDA adolescent requirements, as well as to determine its bioavailability and shelf stability.

## 1 INTRODUCTION

Malnutrition is one of the major concerns of sub‐Saharan African countries such as Uganda (Bain et al., 2013), commonly manifesting among children, women of childbearing age and youth (UNAP, 2016), adolescents being a crosscutting age‐group. Of the estimated 34.6 million people in Uganda by 2014, 55% were below 18 years of age and 22% between 18 and 30 years (UBOS, 2015), implying that a good proportion of these are teenagers/adolescents. During adolescence, there is an increased demand for micro‐ and macronutrient requirements especially energy, iron, and folic acid by girls and zinc and vitamin A for boys (Serra‐Majem, García‐Closas, Ribas, Pérez‐Rodrigo, & Aranceta, 2001). Failure to consume an adequate diet during adolescence results into delayed growth and sexual maturation, cancers, and cardiovascular diseases among other adult diet‐related chronic diseases (Story & Stang, 2005). About half of the adolescent girls living in sub‐Saharan Africa are anemic, iron deficiency being one of the leading contributors to the anemia (Nelima, 2015). Iron deficiency is one of the major causes of YLD’s (years lived with disability) among adolescents (Cherry, Baltag, & Mary, 2016).

A proportion of Uganda’s adolescents (13–21 years) go to boarding schools for three academic terms in year, that is, 3/4 of a year. During this period, they feed on an imbalanced diet consisting of maize meal porridge for breakfast and beans and highly polished maize bread (posho) for lunch and supper, the latter being often replaced with cassava and polished rice (Nabaseruka, 2010). Beans adequately provide protein and energy but inadequate amounts of micronutrients such as vitamin A, iron, and calcium to meet the teenage/adolescent Recommended Dietary Allowance (RDA) requirements (Enyisi, Umoh, Whong, Alabi, & Abdullahi, 2014). The germ and hull of the maize and rice grains, which are often good sources of thiamin, pyridoxine, and phosphorus, and a fair source of riboflavin, niacin, folate, biotin, iron, and zinc are removed from the highly polished maize and rice meal (Slavin, Jacobs, & Marquart, 2000). In addition to being deficient in some nutrients, these foods are disliked by the adolescents and treated as food for the poor and disadvantaged (Pereznieto, Walker, Villar, & Alder, 2011). In an attempt to create variety in their diet and to satisfy their nutritional demands, adolescents resort to energy‐rich but nutrient‐lean snacks sold in school canteens (personal information). This leaves them in a wanting nutritional state, as neither do the foods provided by the school sufficiently cater for a balanced diet (Turatsinze, 2012) nor do the different snacks (usually high in sodium and energy) available to them in school canteens (Valverde, Hernández‐Pérez, & Paredes‐López, 2015).

Snacking illustrates preference for convenience foods due to the limited time to prepare/access full meals among other reasons (Wales, 2017). Hence, convenient foods such as instant nutritious dehydrated soups that require less time to prepare (Kalb, 2012) can be used to improve the nutritional status of adolescents. Dried soup mixes are usually stable against enzymatic and oxidative spoilage and flavor deterioration at room temperature over long periods (over 12 months) without refrigeration (Abdel‐Haleem & Omran, 2014). However, the soup mixes should be adequately nutritious if they are to contribute to the adolescents’ micro‐ and macronutrient requirements. Among plant foods, amaranth has proved a highly digestible and a better source of protein, carbohydrates, and micronutrients such as minerals (calcium, sodium, and potassium) and vitamins A, E, C, and folic acid (Mburu, Gikonyo, Kenji, & Mwasaru, 2012; Mnkeni, Masika, & Maphaha, 2007; Muyonga, Andabati, & Ssepuuya, 2014). Amaranth and other nutritious food ingredients can thus be used to make a nutritious dry soup mix that is convenient to use by adolescents.

Continuous insufficient intake of macro‐ and micronutrients by school going adolescents may result in poor health, reduced physical growth and development, and a decline in educational performance (Ochola & Masibo, 2014). In Uganda, the poor diet is reported to negatively influence academic performance (Nabaseruka, 2010). This, therefore, puts the country at risk of not only producing an educated young population with reduced performance but also perpetuating malnutrition. This study aimed at contributing to addressing this challenge through developing a micronutrient‐rich, amaranth‐based shelf‐stable soup that is acceptable to adolescents and less time‐consuming (convenient) to prepare.

## 2 METHODOLOGY

### 2.1 Soup preparation and reconstitution

**Figure 1:**
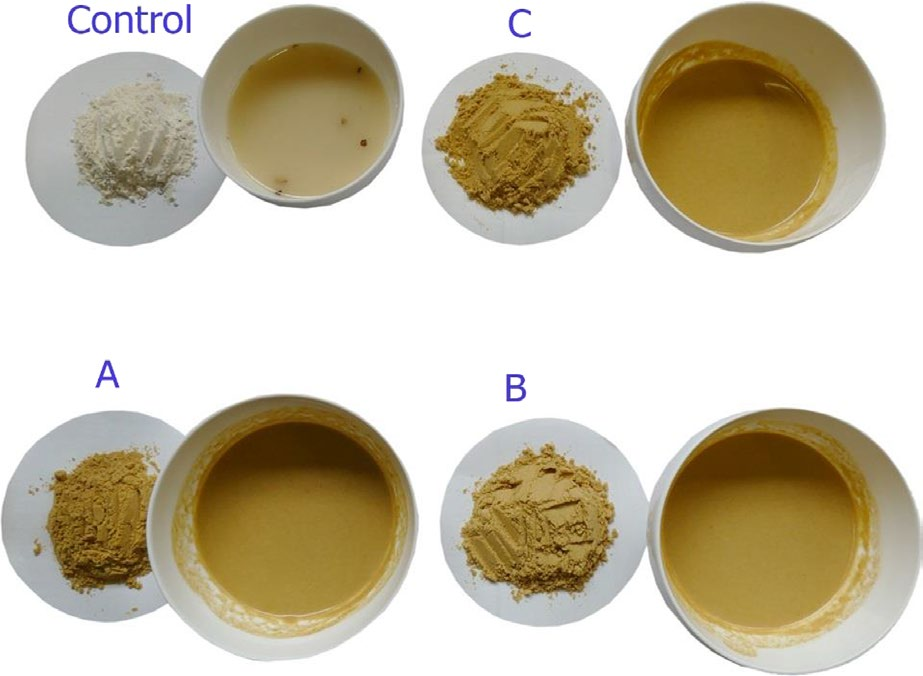
Formulated and control soups

Vegetables that are good sources of target nutrients were chosen as ingredients for formulation of the soup (Table S1). Fresh vegetables (green pepper, onions, and carrots) and potatoes were commercially sourced, washed thoroughly (one volume of fruit washed three times in twice its volume of potable water), disinfected with 200 ppm sodium hypochlorite solution, and thoroughly rinsed. The clean vegetables were blanched in boiling water (95°C) for 10 min, soaked in a 2% brine solution to prevent enzymatic browning, and later cooled. The cooled vegetables were chopped/cut into small pieces that were dried on clean and disinfected trays at 60°C for 8 hr in an air convection dryer (CE TAURO B. Master. 2012, Camisano Vicentino—Italy). The dried vegetables were later ground into a fine flour using an electric grain mill (model 2000; Wonder mill, CA, USA). The ingredient flours were mixed in proportions according to formulations (Table S2) generated by NutriSurvey (2014). Formulations aimed at contributing 25% of the adolescent RDA requirements for carbohydrate, protein, dietary fiber vitamin A, iron, zinc, and calcium. The individual flour proportions were blended in a mixer to obtain uniform mixtures of the formulated soups that were packed in airtight containers. To 15 g of the formulated dry soup, 200 ml of boiling water was added and the mixture stirred for 2–3 min to obtain a nonlumpy cream‐like soup. For the commercial control soup, a 50‐g pack was mixed with 850 ml of cold water in a cooking pan on a heating source and stirred continuously until boiling (Figure 1).

### 2.2 Nutrient, sensory, and functional analyses

For proximate analysis, moisture content was determined by the draft oven method (Nielsen, 2010); crude protein by the Kjeldahl AOAC 2001.11 method (AOAC, 2001; Liu & Rosentrater, 2011); fat content by the Soxhlet method (Nielsen, 2010); total mineral content by ashing in a carbolite furnace at 500°C (Nielsen, 2010); dietary fiber content by the acid detergent fiber assay (Kirk & Sawyer, 1991); carbohydrate content by difference as nitrogen‐free extract (NFE) (Nielsen, 2010); and potassium and phosphorus content by spectrophotometry following wet digestion (Okalebo, Gathua, & Woomer, 2002). Total carotenoid content (total provitamin A compounds) was determined by a spectrophotometric method described in the HarvestPlus handbook of carotenoid analysis (Rodriguez‐Amaya & Kimura, 2004). The total carotenoids content was converted to retinol activity equivalents (RAE) using a conversion of 12 μg provitamin A = 1 μg RAE which is equivalent to 1 μg retinol (vitamin A) (Preedy, 2012). Sensory acceptability was assessed using a 9‐point hedonic scale using an untrained consumer panel of 30 people (Kemp, Hollowood, & Hort, 2009) to evaluate the flavor, thickness, taste, aftertaste mouthfeel, appearance, and overall acceptability. Bulk density, water absorption, and swelling capacity were determined using procedures elaborated by Bamidele, Ojedokun, and Fasogbon (2015), while dispersibility was determined by methods described by Olu et al. (2012).

### 2.3 Statistical analyses

Data were analyzed using IBM SPSS Statistics for Windows (version 16; IBM Corporation, Armonk, New York). Descriptive statistics (means and standard deviations) were derived for the different samples for all attributes of the sensory, nutritional, and functional properties of the formulated and control soups. The effect of formulations on the sensory, functional, and nutritional properties was determined by ANOVA at an alpha value of 0.05, and the means were separated using Tukey’s test. Graphs were generated using MS Excel (version 2010).

## 3 RESULTS

### 3.1 Nutritional composition of the formulated soups

Except for protein content, there was no significant difference among mean macronutrient (carbohydrates, fat, dietary fiber, and ash) content values of the three formulated soups (Table 1). The formulated soups’ energy and protein contents were similar to those of the commercial soup. However, on average, the formulated soup had a dietary fiber (8.17 ± 1.56–10.27 ± 0.40 g/100 g) and fat (10.56 ± 0.29–11.40 ± 0.33 g/100 g) contents that were, respectively, ~8–10 and 3 times higher than that of the commercial soup. On the contrary, the ash content of the commercial soup (16.79 ± 0.04) was twice that of the formulated soups (7.93 ± 0.49–8.75 ± 0.28 g/100 g). The zinc (0.83 ± 0.05–0.87 ± 0.01 mg/100 g) and iron (2.33 ± 0.06–4.37 ± 0.03 g/100 g) contents of the formulated soups were about 4 and 13–25 higher than those of the commercial soup (0.02 ± 0.00 mg/100 g zinc and 0.17 ± 0.02 mg/100 g iron). Basing on the conversion by Preedy (2012) where 1 μg RAE = 1 μg of retinol (vitamin A), the formulated soups had significantly much higher vitamin A content (154.19 ± 12.62–292.68 ± 3.56 μg/100 g) compared to the commercial soup (Table 1).

**Table 1:** Nutrient composition per 100 g of the formulated and commercial soup

Nutrient	Macro‐ (g/100 g) and Micro‐ (mg/100) nutrient			
	A	B	C	CS
Energy (kcal)	320.92 ± 52.42^a^	364.78 ± 5.21^a^	359.39 ± 4.01^a^	326.82^a^
Moisture (g/100 g)	6.06 ± 0.19^a^	5.91 ± 0.15^a^	5.86 ± 0.19^a^	4.89 ± 0.09^b^
Carbohydrates (g/100 g)	52.15 ± 3.37^a^	53.67 ± 0.77^a^	51.20 ± 0.40^a^	60^b^
Protein (g/100 g)	12.30 ± 0.25^a^	13.26 ± 0.25^a^	13.09 ± 0.1^a^	12.95 ± 0.16^a^
Fat content (g/100 g)	10.72 ± 0.26^a^	10.56 ± 0.29^a^	11.40 ± 0.33^a^	3.4^b^
Dietary fiber (g/100 g)	10.00 ± 3.41^a^	8.17 ± 1.56^a^	10.27 ± 0.40^a^	1.0^b^
Ash content (g/100 g)	8.75 ± 0.28^a^	7.93 ± 0.49^a^	8.27 ± 0.28^a^	16.79 ± 0.04^b^
Vitamin A (RAE/μg)	231.25 ± 6.13^a^	154.19 ± 12.62^b^	292.68 ± 3.56^c^	0.53 ± 0.017^d^*
Iron (mg)	2.33 ± 0.06^a^	3.41 ± 0.02^b^	4.37 ± 0.03^c^	0.17 ± 0.02^d^
Calcium (mg)	93.61 ± 3.10^a^	67.82 ± 1.44^c^	73.47 ± 0.65^c^	431.86 ± 0.91^b^
Zinc (mg)	0.85 ± 0.03^a^	0.87 ± 0.01^a^	0.83 ± 0.05^a^	0.02 ± 0.00^b^

“Mean ± standard deviation” values in the same row carrying the same letter as a superscript are not significantly different at *p* > .05.

CS, commercial soup; RAE, retinol activity equivalents; A, B, and C are different versions of the formulated soup.

Values without standard deviations were obtained from the package of the commercial soup.

### 3.2 Sensory acceptability of formulated soups

**Figure 2:**
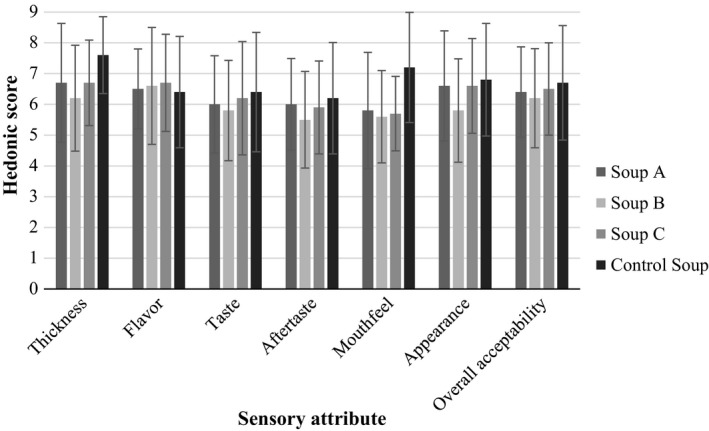
Sensory acceptability of formulated soups

The formulated soups acceptability ranged between 6.0 and 6.7 for all the sensory attributes, except mouthfeel and aftertaste which had scores ranging between 5.4 and 6.0 (Figure 2). Hence, though for all the formulated soups, none of the attributes scored 8 and above (was liked very much and beyond) on the nine‐point hedonic scale, none of them was disliked.

### 3.3 Functional properties of the formulated soups

The swelling capacity (SC) for all the three formulated soups ranged between 5.05 and 5.38 g/g significantly lower than the commercial soup (5.98 ± 0.410 g/g). The bulk density of all the formulated soups ranged between 0.79 and 0.80 g/ml and thus similar but significantly lower than that of the commercial soup (0.84 g/ml) (Table 2).

**Table 2:** Functional properties of the amaranth‐based soup

Sample code	Swelling capacity (g/g)	Bulk density (g/ml)	Water absorption capacity (g/g)	Dispersibility (%)
A	5.33 ± 0.11^b^	0.80 ± 0.002^b^	1.70 ± 0.11^ab^	69.67 ± 0.58^b^
B	5.05 ± 0.18^b^	0.80 ± 0.004^b^	1.83 ± 0.07^bc^	71.33 ± 0.58^c^
C	5.38 ± 0.13^ab^	0.80 ± 0.000^b^	1.98 ± 0.02^bc^	69.33 ± 0.58^b^
CS	5.98 ± 0.41^a^	0.84 ± 0.006^a^	1.53 ± 0.18^a^	77.00 ± 0.00^a^

Mean values in the same column carrying the same letter as a superscript are not significantly different at *p* > .05.

CS, commercial soup; A, B, and C are different versions of the formulated soup.

Water absorption capacity (WBC) for the commercial soup (1.53 ± 0.176 g/g) was significantly lower than that of the formulated soups. The dispersibility of the formulated soups ranged between 69.33% and 71.33%, with the dispersibility of the commercial soup being significantly higher (77.00%) than that of the formulated soups.

### 3.4 Contribution of the amaranth‐based soup toward adolescent RDA requirements

In addition to having comparable functional properties and nutrient composition to other soups, soup C had the highest overall acceptability (Figure 2). It was therefore chosen to illustrate the contribution of the instant amaranth‐based soup to adolescent RDA requirements as stated by Story and Stang (2005). The formulated soup C contributed over 25% of the carbohydrate, protein, dietary fiber, vitamin A, and iron adolescent RDA requirements. This was not possible for zinc, calcium, and energy (Table 3).

**Table 3:** Percentage contribution of the formulated soup to RDA requirements of adolescents 13–19 years of age

Energy/Nutrient	Average RDA (13–19 years)		Percentage contribution RDA	
	Females	Males	Females	Males
Energy kcal/day	2,368	3,152	15.2	11.4
Carbohydrates g/day	130	130	39.4	39.4
Total fiber (g/day)	28	38	36.7	27.0
Protein g/day	46	52	28.3	25.0
Vitamin A (RAE/μg)	700	900	41.8	32.5
Calcium (mg/day)	1,300	1,300	5.7	5.7
Iron (mg/day)	15	11	29.1	39.7
Zinc (mg/day)	9	11	9.2	7.5

RAE, retinol activity equivalents.

## 4 DISCUSSION

### 4.1 Nutritional composition of the formulated soups

The protein content of the formulated soups was similar to the protein content range of amaranth (12% and 13%) on a dry matter basis (Muyonga, Nabakabya, Nakimbugwe, & Masinde, 2008) implying that amaranth was the major contributor. The fat content of the formulated soups was about 10%–11%, twice the amount of oil added during the formulation and significantly higher than the fat content of the commercial soup (3.4%). This could be majorly attributed to pumpkin seeds (powder) whose oil content is above 40% (Murkovic, Piironen, Lampi, Kraushofer, & Sontag, 2004) as well as contributions from other ingredients. The formulated soups were good sources of dietary fiber especially for adolescents whose dietary fiber intake is low (Storey & Anderson, 2014; Vitolo, Campagnolo, & Gama, 2007). In developing countries, adolescents’ diets are characterized by low fruit and vegetable consumption and increasing intake of high‐energy snacks/foods (Ochola & Masibo, 2014) that are often associated with low fiber (Suresh Babu & Sethi, 2005; Unnithan‐Kumar & Tremayne, 2011).

Although the commercial soup’s ash content was higher than that of the formulated soups by about 60%, it was low in iron and zinc, some of the most important minerals in adolescent nutrition (Haider, 2006). This is possible because the commercial soup was not formulated to address the latter. The higher iron and retinoic acid equivalents of sample C are attributable to the larger portion of pumpkin seeds and carrots in the formulation which are, respectively, rich in iron and carotenoids (da‐Silva Dias, 2014; Elinge et al., 2012). The soups’ moisture contents were far below 14%, above which microbial growth and chemical changes during storage can be triggered (Tharise, Julianti, & Nurminah, 2014) and hence expected to be shelf stable. However, due to the presence of oil, there is a possibility of spoilage from oxidative rancidity and its shelf stability should be empirically investigated.

### 4.2 Sensory acceptability of formulated soups

Overall, all the formulated soups had scores similar to that of the commercial soup except for mouthfeel and aftertaste. The low scores of the latter were possibly due to the gritty texture/mouthfeel as expressed by the panelists. The eggshell powder was included as a calcium source (Table S2) but could not solubilize in water during the reconstitution process leading to grittiness. This, therefore, implies that either an alternative calcium source has to be found or better ways of incorporating eggshell powder into the soup should be devised. The acceptability scores of the formulated soups being similar to that of the commercial soup indicate that the former can have a market value similar to that of the commercial soup, especially if the mouthfeel and aftertaste are improved. Never the less, there is a need to improve the sensory acceptability of the formulated soups.

### 4.3 Functional properties of the amaranth‐based soup

#### 4.3.1 Swelling capacity

According to Suresh (2013), the swelling capacity of flours depends on the particle size, the crop variety from which the flour is made, and the types of processing methods or unit operations that the flour undergoes. However, as the unit operations, particle size, and ingredient variety were the same for all soups, the difference in swelling capacity could be attributed to the differences in the amounts of ingredient flours in the formulated soups. Soup B with the highest proportion of amaranth flour had a significantly lower swelling capacity, while soup C with lowest proportion of amaranth had the highest value. This rhymes with observations by Tharise et al. (2014) in which the swelling capacity of the composite flour decreased with increasing proportion of ingredients high in protein, the latter being amaranth in this case. Despite having a similar protein content to the formulated soups, the commercial soup showed a significantly higher swelling capacity. This is possibly because the flour from which the commercial soup was made was finer than the flour from which the formulated soups were made, as swelling capacity reportedly increases with a decrease in particle size (Rao et al., 2016). The fineness of the flour (small particle size) is associated with improved viscosity and smoothness of paste (Kaur, Oberoi, Sogi, & Gill, 2011) features that were observed upon reconstitution of the formulated soup. This however could be improved by increasing the fineness of the formulated soups.

#### 4.3.2 Bulk density

This depends on the particle size and initial moisture content of flours (Suresh, 2013) which were similar for all the formulated soups. Small particle size is associated with higher bulk density (Anandharamakrishnan, 2017) which explains why the finer commercial soup had a higher bulk density than the formulated soups. It is also an important parameter in packaging and shipping considerations as it can be used to predict how much of the material, by weight, will fit into a container of specified volume (Finney, Buffo, & Reineccius, 2002). According to Suresh (2013), a high bulk density value of flour suggests its suitability for use in food preparations because a very small quantity can provide substantial functionality. On the other hand, the low bulk density would be an advantage in the formulation of complementary foods because a small quantity is required to attain the required bulkiness of the food product (Suresh, 2013). Flours with low bulk density tend to have higher amounts of occluded air that may favor oxidation and other deteriorative reactions (Anandharamakrishnan, 2017). Hence, although a high bulk density is desirable for functionality, this should not negatively influence shelf stability.

#### 4.3.3 Water absorption capacity (WAC)

The water absorption capacity is important for the quality and texture of food products because it stabilizes the food against undesirable effects such as syneresis (Kaur et al., 2011). Water absorption capacity of flours represents their ability to associate with water (reconstitute) under limited water conditions (Adejuyitan, Otunola, Akande, Bolarinwa, & Oladokun, 2009; Suresh, 2013). High WAC values are associated with better reconstitution abilities of powders (Tharise et al., 2014) which implies that the commercial soup’s ability to reconstitute was lowest compared to the formulated soups. The observed range (1.53 ± 0.175–1.98 ± 0.015 g/g) is similar that of wheat (1.96 g/g), rice (1.92 g/g), and potato flours (1.40 g/g) (Suresh, 2013). There is no published information on WAC of composite instant flours, but this observed range showed good reconstitution abilities.

Dispersibility is the ease with which the powder becomes distributed as single particles in the bulk liquid phase (Shittu & Lawal, 2007). The higher the dispersibility, the better the powder/flour reconstitutes in water and gives a fine constituent during mixing (Oluwole et al., 2016). The dispersibility of the formulated soups ranged between 69.33% and 71.33% which was similar to that observed in the yam flour (Obadina, Babatunde, & Olotu, 2014). However, it was significantly different from the commercial soup’s dispersibility (77%), the latter being similar to that of sorghum–wheat composite flour 73.5% and 76.5% (Adebowale, Adegoke, Sanni, Adegunwa & Fetuga, 2012) both of which showed good reconstitution abilities. This, therefore, calls for improving the dispersibility of the formulated soup to above 73.5% for which good reconstitution has been observed.

### 4.4 Contribution of the amaranth‐based soup toward adolescent RDA requirements

The soup was able to meet the target 25% contribution to the carbohydrate, protein, dietary fiber, vitamin A, and iron RDA requirements for adolescents. However, there is need to reformulate the soup with a purpose of increasing the amounts of energy, calcium, and zinc to meet the target 25% contribution. However, reformulation should not negatively affect the functional and sensory properties of the soup. Calcium in adolescents is required to cater for the rapid expansion of bone mass while energy is required to facilitate the increased metabolism resulting from the increased growth rate during adolescence (Mcguire & Beerman, 2016; Shils, Maurice, & Shike, 2006). In developing countries, adolescents’ intake of energy and micronutrients is low (Ochola & Masibo, 2014) and such convenient products can contribute to addressing the problem. Snacking should not be discouraged but, rather, adolescents should be encouraged to improve their food choices during snacking to more nutrient‐dense foods (Brown, Isaacs, Krinke, Lechtenberg, & Murtaugh, 2010). However, the product’s ability to meet RDA requirements is itself unimportant if the nutrients are not available for use by the body (Caballero, Allen, & Prentice, 2005). For example, it takes about 30 min for calcium in eggshells to dissolve in water at 70°C (Dolińska, Jelińska, Szulc‐Musiok, & Ryszka, 2016; Eskin & Shahidi, 2012) yet only 2–3 min are used for reconstituting the soup, thus being insufficient for dissolving all the calcium. Limited solubility may limit its availability for assimilation into the body. It is hence important to ensure that the micro‐ and macronutrients are bioavailable to the adolescents’ bodies upon intake.

## 5 CONCLUSION

The study succeeded in developing an acceptable instant nutritious amaranth‐based vegetable soup. The soup demonstrated ability to contribute to over 25% of the required RDA macro‐ and micronutrient requirements for adolescents aged 13–19 years, especially carbohydrate, protein, dietary fiber vitamin A, and iron. The formulated soup displayed good instant properties with a preparation time of 5–10 min that properly fits within the time allocated for meals in secondary schools. This convenience shall stimulate its use and enable adolescents meet their RDA requirements. If adopted, components such as eggshells and vegetables that could not be consumed fresh can be transformed into shelf‐stable products thus cutting down postharvest losses and contributing to increased nutrition security among adolescents.

## 6 RECOMMENDATION

Further research should be conducted to (i) increase acceptability of the soup and alter proportions of ingredients to increase the amounts of zinc and calcium to meet the target contribution of 25% to adolescent (13–19 years) RDA requirements without reducing acceptability and functionality; and (ii) determine the effect of processing conditions (blanching and drying temperature) and storage on nutrient bioavailability.

## CONFLICT OF INTEREST

The authors declare that they do not have any conflict of interest.

## ETHICAL REVIEW

This study does not involve any human or animal testing.
