# Supplementary material for: Contribution of instant amaranth (Amaranthus hypochondriacus L.)‐based vegetable soup to nourishment of boarding school adolescents
Source: Food Sci Nutr. 2018 Jun 6;6(6):1402–9. doi: 10.1002/fsn3.664 (PMC6145273; doi:10.1002/fsn3.664)
Supplement: Supplementary file 1 [file FSN3-6-1402-s001.pdf]

Supplementary material

Table S1. Choice of ingredients for target nutrients

**Ingredient**	**Target micronutrient/ functional purpose**	**Amount**	**Reference**
Carrots	Vitamin AREq(μg)	1295.22	Fabiyi et al. 2012
Egg shells	Calcium(g)	98.2 -100	King'Ori, 2011
Green pepper and Onions	Spices. (flavonoids)	N/A	Dias, 2012
Grain amaranth	vitamins A(μg), calcium(mg/g), and iron(mg/g)	4656, 175 and 17.4	Mulokozi et al. 2004; Muyonga et al. 2008
Pumpkin seeds	Iron and zinc(mg/100g)	14.14 and 3.75	Elinge et al. 2012
Irish potato	source of starch (thickener)	15-25 %	Harry et al. 1966
Hydrogenated vegetable oil	Coating (hydrophobic) properties	5 % (W/W)	Abeysinghe & Illeperuma, (2010)

Table S2: Percentage composition of the Nutri-survey formulated soups

Ingredient	% composition		
	A	B	C
Grain amaranth	53	55	50.0
Potato powder	17	17	16.5
Pumpkin seeds	11.5	10	15.2
Egg shells	3.8	3.7	3.7
Carrots	3.8	3.6	4.0
Onions	4.3	4.0	4.0
Green Pepper	1.1	1.1	1.1
Hydrogenated vegetable oil	4.0	4.0	4.0
Salt	1.5	1.5	1.5
